# Multifamily Therapy in Adult Psychiatry: Forgotten Origins, Proven Evidence, and Institutional Barriers

**DOI:** 10.1192/j.eurpsy.2026.11741

**Published:** 2026-07-31

**Authors:** O. Amiot, E. Petiau, D. Waintrater, L. Koziatek Molina, I. Fradi, M. C. Beaucousin

**Affiliations:** 93, Etablissement Public de Santé Ville Evrard, Saint ouen, France

## Abstract

**Introduction:**

Multifamily Therapy (MFT) was born in adult psychiatry more than 60 years ago, when Peter Laqueur first gathered families of patients with schizophrenia in public hospitals (Laqueur, *Multi-family group therapy.* Basic Books 1964). At the time, involving relatives was revolutionary, acknowledging that illness affects the entire family. Yet today, MFT is far more common in child psychiatry, where it is applied to eating disorders, anxiety, and neurodevelopmental conditions, while remaining rare in adult services.

This paradox invites us to question our practices: why has adult psychiatry largely abandoned MFT, despite strong evidence of its effectiveness?

**Objectives:**

This paper explores the institutional, cultural, and organizational barriers that have prevented the dissemination of MFT in adult psychiatry, despite strong clinical evidence supporting its benefits for schizophrenia, bipolar disorder, and first-episode psychosis.

**Methods:**

A narrative review of international literature and clinical reports was conducted, focusing on barriers identified in adult services and contrasted with evidence of MFT effectiveness. Sources included controlled trials, guidelines, and qualitative evaluations.

**Results:**

Three main barriers emerge.

1. **Biomedical dominance:** adult psychiatry remains focused on symptoms, relapses, and pharmacological stabilization, reducing patients to diagnoses and leaving little space for relational interventions.
2. **Institutional fragmentation:** children, parents, and siblings are often treated in separate, uncoordinated services, with no shared narrative of family experience. This structural division discourages systemic practices.
3. **Daily obstacles:** engaging families whose ties may be fragile, finding trained staff, and legitimizing group interventions within rigid institutional cultures.

These obstacles persist even though family interventions are internationally recognized as effective in preventing relapse, reducing caregiver burden, and improving communication (McFarlane, *Family Process* 2016; 55: 460–482; NICE, *Guideline* 2014; Gleeson et al., *Early Interv Psychiatry* 2025; in press). The gap between evidence and implementation reflects organizational inertia rather than lack of efficacy.

**Conclusions:**

MFT is an evidence-based, systemic intervention whose marginalization in adult psychiatry is due less to clinical limitations than to institutional barriers. Reviving MFT requires rethinking service organization, legitimizing family as a therapeutic resource, and supporting professionals through training and cross-sector collaboration. Reintegrating MFT in adult psychiatry would not only improve clinical outcomes but also reconnect services with their systemic roots. It could also act as a lever for cultural change, shifting care models from a narrow biomedical focus towards a relational, recovery-oriented psychiatry.

**Disclosure of Interest:**

None Declared

